# Ten years of exploration, a new journey to start: advancing *Chinese Medicine* to the next level

**DOI:** 10.1186/s13020-017-0147-8

**Published:** 2017-09-21

**Authors:** Chunming Wang, Chin Kio Lei, Ruibing Wang, Jianbo Xiao, Yitao Wang

**Affiliations:** 1 Editorial Office (Macau) of Chinese Medicine, International Society for Chinese Medicine, Macau, China; 2 State Key Laboratory of Quality Research in Chinese Medicine, Institute of Chinese Medical Sciences, University of Macau, Macau, China

The past decade has witnessed the rapidly growing influence of Chinese medicine around the world. Both basic and clinical research into Chinese medicine has continued flourishing and become more international, interdisciplinary and interactive than ever. It was such a good timing that *Chinese Medicine* (‘the Journal’)—the official journal of the International Society for Chinese Medicine (‘the Society’) [1]—celebrated its tenth anniversary last year (2006–2016). Over the past decade, the Journal has grown from scratch into one of the most reputable journals in the field of complementary medicine, proudly serving a broad audience of researchers, practitioners, educators, learners and anyone else interested in Chinese medicine. In 2016, we at the Society concluded a 2-year review of the Journal’s development. We had analysed our strategies and achievements, identified challenges and opportunities, consulted our valued authors, reviewers, fellow editors and frontline researchers, and analysed the feedback and suggestions (both official and casual) from the community. The outcomes of this review convinced us that it would be the time for revamping. The Society soon announced to implement a series of strategic reforms, aimed at improving the management and development of the Journal and better serving the global community of Chinese medicine.

First of all, the Society is delighted to announce the inauguration of the new leadership of the Journal, including the new Editor-in-Chief Professor Yitao Wang, six new Associate Editors, an international editorial and senior advisory board, as well as a Macau-based Editorial office. The Society sincerely thanks the former editorial and advisory team for their past service and hard work that has shaped our journal into what it is now; without their vision, leadership and dedication, the journal would hardly have made such achievements. Now, the new team comprises both established and emerging scholars from across the globe and interacts with a large pool of young, active scientists—they have already started to contribute their expertise in the past few months. The new team, together with our publishing partner BMC, pledges to bring the Journal into a new era, enabling it to evolve while firmly upholding its long-standing mission and strengths.

Next, the new editorial office would like to highlight three specific changes, among other ongoing renovations, which are crucial for the interest of our readers, authors and reviewers.

## Accelerating the publication of manuscripts

We acknowledged that it is necessary to shorten the manuscript processing time, based on the data in the past 3 years. The speed of publication (and rejection) is important to both our authors and the Journal. We strive to persist in the founding vision of the Journal—‘publishing evidence-led, scientifically justified, and ethical research into Chinese medicine’. [2] Yet, changes are necessary to increase efficiency. Timely publication of scientific discoveries builds up a favourable reputation of a journal and is extremely helpful to the authors, whose next research paper, funding proposal or business plan will be built upon the publication of the present finding. A journal known for sluggish processing of manuscripts and vague instructions to authors can hardly attract good research papers from serious scientists but will only frighten off the audience and compromise the journal’s reputation.

We aim to make decisions on manuscripts faster using several approaches, which we have already started since March 2017. First, we strengthen our editorial screening before peer-review, with the help from the new international editorial team, so as to save time for authors whose papers are not falling into the scope of our journal or significantly lack scientific soundness. Second, in addition to the existing reviewers who have long helped us, we create a larger pool of reviewers including both established scientists and emerging talents, based on a strong network and rigorous recommendation from our editorial and advisory boards. Third, we cancel the post-acceptance editorial process, based on the feedback from many past authors and our own analysis into the purpose of scientific publishing.

We are committed to shortening the waiting period for authors, in spite of the generally long publication process in biomedical journals, according to a recent analysis published in *Nature* [3]. We highly appreciate the patience of our authors during the past years and believe *Chinese Medicine* will be a leading journal in processing efficiency in the near future. This should be achieved without sacrificing the quality of peer review and the publishing standards we hold on to. We will show zero tolerance to a manuscript with ethical or research integrity-related issues.

## Actively supporting authors in submission and publication

We are dedicated to collaborating with the authors in publishing good research. And it will be our collaborative efforts to maintain research integrity. First, we have recently established a two-step similarity detection system to check each new submission, to help authors avoid the use of excerpts or complete sentences from other sources. We believe that deliberate plagiarism is rare, but we found this year a couple of cases that the authors unintentionally wrote the paragraphs that they previously published in their own paper (‘self-plagiarism’). We are confident that our system is effective in helping the authors rectify such practices.

Second, under the guidance of COPE and with the support from BMC, we continue to emphasise the importance of research ethics to our respected authors and reviewers. We help the authors in completing the Checklist, with an emphasis on the items relating to animal/human subjects. We request authors to submit approvals from human/animal ethics committees for any research conducted with human and/or animal subjects—no matter it is a hypothesis-driven comprehensive research, a survey, a laboratory study involving transgenic mice or a multi-centre clinical trial on patients. A Trial Registration Number should also appear in the abstract of the paper [4], which is a proof of ethical approval but this has often been overlooked.

Third, in response to authors’ feedback that we have received, the Society is now discussing how we can more actively and efficiently support researchers who are planning to submit their best research to the Journal. Existing and potential initiatives include covering the article-processing charge for authors who are in reasonably special circumstances, who are long-term reviewers for the Journal, or who have been involved in organising workshops for the Journal, etc.

## Creating platforms for enhancing two-/multi-way communication

Communication between authors and editors is vital. Hearing no response from the editorial office for long may frustrate the authors; likewise, receiving a revised manuscript with no effective response to the editors’/reviewers’ comments can prevent the editors from making timely decisions. Hence, our new editorial office welcomes enquiries, suggestions and feedback from our authors and is committed to responding as fast as possible.

Meanwhile, we fully understand that reviewers are always busy and highly appreciate the efforts from our past and present reviewers. To invite more experts from different corners of the world to contribute as reviewers, we are promoting the Journal in multiple ways and expanding the expert pool through connections. For example, we have recently established strategic collaborations with academic organisations both in the field of Chinese medicine and beyond the traditional scope—notably including the Phytochemical Society Europe and Phytochemical Society Asia. The Society has co-organised 2nd International Symposium on Phytochemicals in Medicine and Food (2-ISPMF, 2017) held in April 2017 in Fuzhou, China and 3rd International Conference on Natural Products Utilisation: from Plant to Pharmacy Shelf (ICNPU 2017) recently held in Bulgaria. Moreover, the Society is preparing for 3rd International Symposium on Phytochemicals in Medicine and Food (3-ISPMF) to be held in Aug 2018 in Kunming, China. The Journal served as the designated platform to publish the proceedings. We have also organised two rounds of publishing workshop in Macau, having invited chief or associate editors of over 10 sister-journals in the area of pharmaceutical sciences to enhance collaboration in publishing, reviewing and resource-sharing. Additionally, through the strong network of our editorial/advisory board members, we have welcomed increasing numbers of early-career scientists to join our reviewer’s pool and planned to hold a young scientists referee meeting soon. We also prepare to invite the most outstanding ones from them to replenish our editorial board on a regular basis. All these initiatives aim to strengthen the connection between our editorial office and the authors and reviewers of the Journal, which will further benefit the development and promote the reputation of the Journal.

In addition to the above three aspects, much work remains to be done in the coming year. To reiterate, we strive to work hard and smart to advance the Journal to the next stage, while upholding its long-standing mission and commitment to the global academic community. We plan to refine/redefine the scope of the Journal in 1 year, during which we will carefully identify our position in the fields, so as to better serve the authors in different specialities. We understand the importance of Impact Factor to research communities we serve. At the same time we will work with the journal publisher to present more metrics for measuring the impact of the research we publish. Journal gained a 2016 Impact Factor (IF) of 1.508; in the same year, it published 49 research and review articles. We are confident to see the IF increase because of all the efforts mentioned above and the level of commitment from the Editorial Board. Also, we will deepen our collaboration with both our publisher BMC and the organisations across different fields to raise the visibility of the Journal in both academia and the general public. Finally, we would love to welcome suggestions and feedback; we look forward to communicating with you—which is also the principal aim of this editorial article.
